# Doctor, when can I drive? – compensation capability while driving with restricted elbow – a biomechanical analysis

**DOI:** 10.1016/j.jseint.2024.09.028

**Published:** 2024-11-01

**Authors:** Erik Schiffner, Felix Lakomek, Falk Hilsmann, Dominique Schoeps, Max Prost, Christoph Beyersdorf, Joachim Windolf, David Latz

**Affiliations:** Department of Orthopedic and Trauma Surgery, University Hospital, Düsseldorf, Germany

**Keywords:** Driving fitness, Forensic medicine, Elbow, Osteoarthritis, Traffic medicine, Trauma surgery, Compensation, Biomechanics

## Abstract

**Background:**

Every joint participates in a specific range of motion (ROM) while operating a motor vehicle safely. In current literature, there is a paucity of how movement restrictions of the elbow flexion and extension can be compensated by adjacent joints to ensure safe driving. The aim of this study was to analyze movement patterns of the kinematic chain consisting of wrist, elbow, and shoulder while driving with restricted elbow joint.

**Methods:**

Twenty participants completed a driving course in a driving simulator in two conditions: a) free ROM of all joints vs. b) restricted right elbow in 90° flexion but with free pronation and supination. To evaluate driving performance, speed, lane accuracy, and shifting time was measured. To analyze the movement pattern, ROM of wrist, elbow, and shoulder were recorded using a full-body motion capture system. Each driving course consisted of three maneuvers, as follows: I shifting, II left turns, and III right turns. Driving performance and movement patterns of condition a) and b) were compared on maneuver I-III.

**Results:**

Driving performance: Participants drove their car slower while driving right turns with elbow restriction (a) 37.45 ± 1.66 km/h vs. b) 32.53 ± 1.18 km/h; *P* = .02). Driving performance was not affected while driving left turns or shifting gears (*P* > .05). Movement pattern: Participants used their right shoulder in a higher ROM while driving turns with restricted right elbow (*P* < .05) but the left arm showed no significant different movement pattern (*P* > .05). The ROM of the left elbow and both shoulders were significantly higher when shifting gears with restricted right elbow (*P* < .05).

**Conclusion:**

This study first describes the changes in movement patterns of the upper extremity while driving with a restricted right elbow. Our data suggest that restricted right elbow flexion or extension can be compensated by the left arm and a different posture of the right shoulder when driving left turns. A different movement pattern of the left elbow and both shoulders is used when changing gears while driving straight. Drivers should be aware when driving turns while shifting gears, and special attention should be paid to the shoulders and left elbow when evaluating the driving capability of patients with movement restriction of the right elbow by physicians.

Driving a car provides people with personal mobility and autonomy.^8^ The core task of the orthopedics and trauma surgeons is to restore and maintain patients’ mobility. Especially for the elderly or people with disabilities, often driving is the only opportunity to maintain their personal mobility, and an inability to drive results in socio-economic implications.^2^

To safely operate a motor vehicle, it requires a certain amount of strength, dexterity, and range of motion (ROM) of each joint.^3^^,^^4^ In previous studies, joint ROM of the upper and lower extremities that is commonly used while driving a car was defined on healthy subjects.^15^^,^^17^^,^^18^^,^^19^ This information may assist the orthopedics when evaluating a patient’s driving capability.

However, many people feel able to continue driving even if the ROM of one joint is restricted and smaller than that were previously defined.^15^^,^^17^^,^^18^^,^^19^^,^^27^ Thus, it seems possible that movement restrictions of one joint can be compensated to a certain degree by capacity of adjacent joints. To the author’s knowledge, no study has analyzed changes of movement pattern when driving with restricted joints. Thus, no study has analyzed compensation mechanisms of the upper extremity as a functional unit (wrist, elbow, and shoulder) while driving a car.

Previous studies suggest that movement restriction of the elbow impairs driving performance, but movement restrictions of the wrist had no perceptible effect on driving ability.^29^ Thus, the ROM of the elbow seems to be essential for driving safely. However, in current literature, there is a paucity of how someone compensates different kind of movement restrictions.

The aim of this study was to analyze the kinematic chain of wrist, elbow, and shoulder while driving with restricted elbow joint.

## Materials and methods

This is a basic experimental study. There is a positive vote from the ethics committee (2021-1336). Prior the procedure, an informed consent was obtained, and each participant completed a standardized questionnaire, including the Arnett inventory of sensation seeking to analyze the risk behavior of each participant. Only healthy volunteers who drive their own car at least 5000 km/year for the last three years were included. In this study, only twenty right-handed and healthy subjects (12 males, 8 females, 28.32 ± 3.18 years (mean age ± SD)) who drive their own car at least 5000 km/year for the last three years were included. Participants who documented injuries or any other functional disorders regarding knee or ankle were excluded from the study.

### Motion capturing

**Figure 1:**
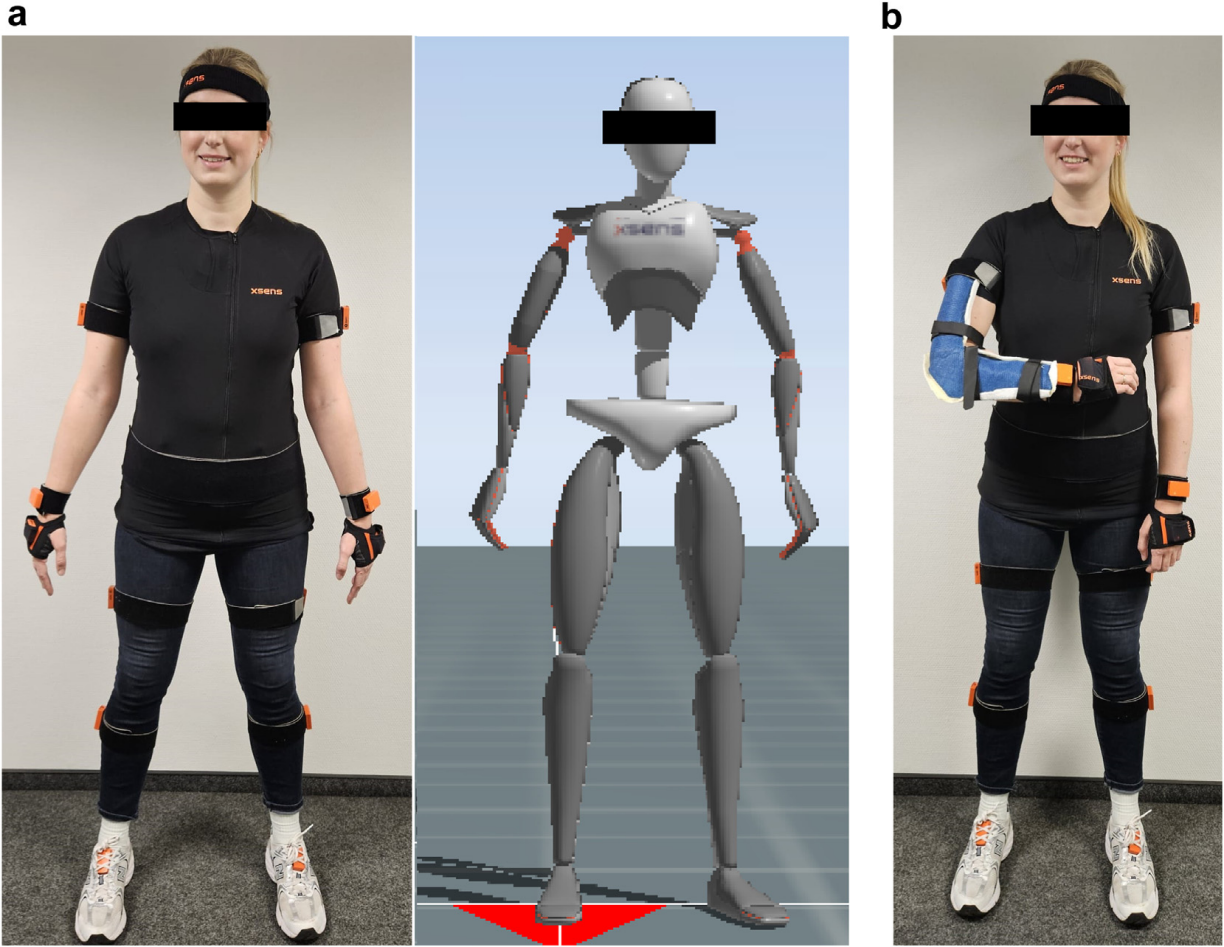
(**a**) To allow a simultaneous measurement of the wrist, elbow, shoulder, spine, and hip, the anatomy of each participant was measured and motion capturing suits and trackers were attached in a standardized manner to create an individual avatar. A connection to a mobile computer system was established using Bluetooth. (**b**) To simulate a fully restricted elbow joint, individual upper arm splints were made to 90° flexion.

First, a detailed individual body anatomy of each participant was measured. For motion analysis, each participant was equipped with a motion capturing suite (Xsens, Xsens Technologies B.V., Enschede, Netherlands; Rehagait Analyzer Pro, Hasomed GmbH, Magdeburg, Germany) and motion trackers were positioned in a standardized manner to create an individual avatar (Fig. 1, *A*). This setup allows monitoring ROM and compensation mechanisms in high resolution (30 hertz), and artifacts can be detected instantly.

To evaluate the compensation capability when driving with an immobilized right elbow, only motion tracker of the wrist, elbow, and shoulder were taken into further analysis. To simulate a restricted elbow joint, individual upper arm splints were made to 90° flexion but with free pronation and supination (Fig. 1, *B*).

### Driving simulation

**Figure 2:**
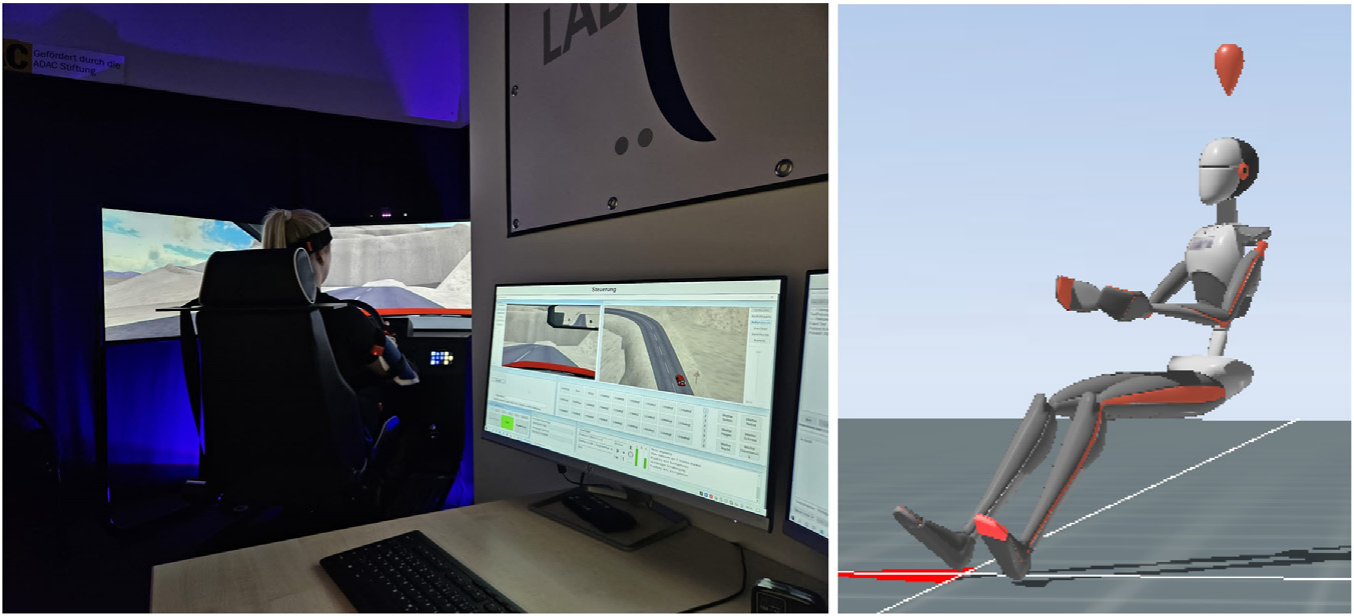
This experimental setup allows monitoring range of motion and compensation mechanisms in high resolution (30 hertz), and artifacts can be detected instantly while driving a car.

After creating an individual avatar and checking for artifacts, all participants were seated in a uniform and standardized position in a driving simulator (Typ Trainer; Foerst Fahrsimulatoren GmbH, Wiehl, NRW, Germany). Left-hand drive simulator was used driving on the right-hand side of the road. Participants were seated with a minimum distance to the steering wheel of 25-30 cm, an as small as possible head-headrest distance and in an as upright as possible suitable backrest inclination.^1^ Before participants start to drive, the attachment of each sensor and stable Bluetooth connection to the computer system was carefully checked (Fig. 2).

After getting familiar to the driving simulator in a three-minute free driving scenario, each participant completed a standardized driving course: a) without any movement restrictions (free ROM) and b) with an elbow splint (elbow restriction) on the right side with free pronation and supination but to 90° flexion restricted elbow (Fig. 1, *B*). A randomized order for a) and b) for each participant was chosen to minimize learning effects. Every participant was instructed to drive as fast but also as accurately as possible but not to exceed a maximum speed of 50 kilometers per hour (km/h). Driving simulation took place in right-hand traffic. Each course consisted of three fundamental maneuvers:

I shifting

II left turns

III right turns

In I, participants had to shift gears up into the third gear while accelerating up to a speed of 50km/h. In II and III, participants had to drive a parkour with three right and left curves (90°-180°) with a car with automatic transmission.

### Data analysis

Data of the motion capturing system and the driving simulator were synchronized and further processed using Excel (Microsoft Corp., Redmond, WA, USA) and SPSS Statistics version 29 (version 29.0.1; IBM Corp., Armonk, NY, USA). Baseline errors were measured to document the accuracy of the testing device. The change of the position of the sensor on the right arm (before or after the cast is placed) does not change the measurement

After the visualization of data and exclusion of any artifacts, statistical analysis was performed:

#### Driving performance

For maneuver I shifting, speed (minimum, mean, and maximum in kilometer per hour (km/h)), lane accuracy (minimum, mean, and maximum distance to the center of the lane in meter (m), left deviation (−), right deviation (+)), and shifting time (from start to third gear in seconds (s)) were measured to determine the driving performance. For maneuver II, left turns and for maneuver III, right turns, speed, and lane accuracy were measured to determine the driving performance. To evaluate compensation capability, the performance with a) free ROM and b) with elbow restriction was compared.

#### Movement pattern

To analyze the movement pattern and compensation capability, ROM (arithmetic mean, maximum, and minimum) of the following joints were measured when driving:

- wrist (flexion, extension, ulnar deviation, or radial deviation)
- elbow (flexion, extension, pronation, or supination)
- shoulder (abduction, adduction, external, internal rotation, ante, or retroversion)

The ROM of the joints was compared when driving a) with free ROM and when driving b) with elbow restriction.

The aim of our statistical analysis was to examine the impact of elbow ROM restriction on driving performance and compensation mechanisms of the adjacent joints, while accounting for variability across the subjects. Given the hierarchical structure of our data, with multiple observations per participant, a linear mixed model was deemed appropriate to account for the potential nonindependence of observations within each participant. The model was specified with a fixed effect for elbow restriction and a random intercept, the model parameters were estimated using restricted maximum likelihood method. Based on estimated marginal means, additional pairwise post hoc comparisons were performed to investigate the differences between the groups (elbow restriction or free ROM, maneuver I-III).

## Results

In this study, twenty healthy subjects participated (12 males, 8 females, 28.32 ± 3.18 years (mean age ± SD)). There was an average Arnett inventory of sensation seeking of 4863 ± 4.07 (mean ± SD). No data had to be excluded due to artifacts.

### Driving performance

The driving performance of a) free ROM and b) elbow restriction was compared on maneuver I-III (Table I). No accident happened.

**Table I:** Speed (km/h), lane accuracy (distance to the center of the lane (m), left deviation (−), right deviation (+)), and shifting time (from start to third gear (s)) were measured to determine the driving performance.

		Free ROM		Elbow restriction		*P*
I Shifting						
Speed	Mean	37.05	±1.11	36.72	±1.47	.68
	Max	47.30	±1.29	47.00	±0.30	.80
Lane accuracy	Min	−0.82	±0.11	−0.76	±0.12	.64
	Mean	−0.49	±0.10	−0.37	±0.10	.20
	Max	−0.12	±0.06	0.17	±0.08	.54
Shifting time	Mean	9.94	±0.46	1.00	±0.39	.83
II left turns						
Speed	Mean	30.31	±0.83	30.29	±0.64	.98
	Max	42.16	±1.19	41.92	±1.01	.86
Lane accuracy	Min	−3.26	±0.25	−3.24	±0.20	.94
	Mean	−1.73	±0.15	−1.75	±0.15	.88
	Max	0.14	±0.13	0.17	±0.11	.78
III right turns						
Speed	Mean	22.54	±0.78	22.14	±0.75	.56
	Max	**37.45**	±1.66	**32.53**	±1.18	**.02**
Lane accuracy	Min	−2.16	±0.30	−1.83	±0.12	.24
	Mean	−0.19	±0.14	−0.23	±0.13	.77
	Max	1.26	±0.16	1.02	±0.16	.11

ROM, range of motion.

Bold indicates significant value.

For maneuver I shifting, statistical analysis revealed no significant effect for speed, lane accuracy, and shifting time. For maneuver II left turns, the statistical analysis revealed no significant effect for speed and lane accuracy. For maneuver III right turns, the statistical analysis revealed that participants drove their car in a significant lower maximum speed while driving with elbow restriction right turns (*P* < .05, Table I).

### Movement pattern

The movement patterns (arithmetic mean, maximum, and minimum) of the wrist, elbow, and shoulder when driving with a) free ROM and b) elbow restriction were compared across maneuver I-III (Table II).

**Table II:** Wrist flexion° or extension°, ulnarduktion° or radialduktion°, elbow flexion° or extension°, pronation° or supination° and shoulder abduction° or adduction°, external rotation° or internal rotation°, and flexion° or extension° were measured to determine the driving performance.

				Free ROM		Elbow restriction		*P*
I shifting								
Wrist	Right	Flexion (+)/extension (−)	Min	−44.10	±1.92	−39.00	±1.84	.02
			Mean	−31.02	±2.25	−29.22	±2.05	.41
			Max	−8.75	±2.00	−15.70	±2.19	<.01
Elbow	Right	Pronation (>90°)/supination (<90°)	Min	72.15	±3.02	78.15	±3.34	.09
			Mean	93.96	±3.77	85.40	±3.32	.04
			Max	113.80	±3.29	91.10	±3.50	<.01
	Left	Pronation (>90°)/supination (<90°)	Min	77.75	±3.53	68.10	±4.13	<.01
			Mean	87.02	±3.55	81.49	±3.55	.22
			Max	92.90	±3.49	92.00	±2.94	.72
		Flexion (+)/extension (−)	Min	44.60	±3.74	46.75	±3.76	.47
			Mean	49.10	±3.53	59.65	±3.13	<.01
			Max	56.80	±3.38	81.35	±3.97	<.01
Shoulder	Right	Abduction (+)/adduction (−)	Min	10.60	±2.10	15.75	±1.46	.02
			Mean	18.46	±1.79	26.74	±1.33	<.01
			Max	29.70	±1.60	46.55	±1.38	<.01
		External rotation (+) or internal rotation (−)	Min	−3.90	±3.44	−24.10	±2.99	<.01
			Mean	8.84	±2.79	−2.90	±1.98	<.01
			Max	17.65	±2.58	14.80	±2.50	.45
		Flexion (+) or extension (−)	Min	22.50	±1.72	11.40	±1.64	<.01
			Mean	36.47	±1.76	33.74	±2.21	.12
			Max	47.55	±1.60	47.85	±2.76	.88
	Left	Abduction (+) or adduction (−)	Min	1.75	±1.73	5.25	±1.13	.03
			Mean	4.46	±1.51	9.56	±1.21	<.01
			Max	8.10	±1.28	16.20	±1.56	<.01
		External rotation (+) or internal rotation (−)	Min	16.15	±2.44	8.25	±2.66	<.01
			Mean	21.19	±2.75	16.41	±2.27	<.01
			Max	24.60	±2.70	23.50	±2.50	.64
II left turns								
Wrist	Right	Flexion (+) or extension (−)	Min	−44.70	±2.80	−35.75	±5.25	<.01
			Mean	−27.90	±2.36	−24.57	±2.44	.09
			Max	−3.80	±3.22	−5.25	±2.53	.56
Elbow	Right	Pronation (>90°) or supination (<90°)	Min	46.60	±12.77	80.05	±3.65	.03
			Mean	99.60	±4.43	88.01	±3.90	<.01
			Max	125.25	±4.90	95.15	±4.26	<.01
Shoulder	Right	Abduction (+) or adduction (−)	Min	−3.90	±2.46	9.05	±1.92	<.01
			Mean	5.36	±2.39	16.14	±1.83	<.01
			Max	17.95	±2.96	22.85	±1.85	.08
		Flexion (+) or extension (−)	Min	28.20	±2.99	33.55	±3.05	.04
			Mean	48.91	±3.17	47.22	±3.44	.6
			Max	64.00	±2.39	62.35	±3.89	.69
II right turns								
Wrist	Right	Flexion (+) or extension (−)	Min	−48.60	±2.04	−35.15	±3.05	.01
			Mean	−26.39	±2.14	−21.61	±2.16	.01
			Max	−8.25	±4.37	−1.45	±2.82	<.01
Elbow	Right	Pronation (>90°) or supination (<90°)	Min	45.20	±3.50	74.55	±3.91	<.01
			Mean	75.32	±3.55	82.09	±3.78	.03
			Max	110.45	±4.16	91.20	±3.75	<.01
Shoulder	Right	Abduction (+) or adduction (−)	Min	4.20	±2.20	12.90	±1.58	<.01
			Mean	12.22	±2.12	18.81	±1.68	<.01
			Max	22.25	±1.98	26.15	±1.85	.10
		External rotation (+) or internal rotation (−)	Min	−0.05	±3.89	−3.95	±3.14	.42
			Mean	12.14	±3.40	5.61	±3.02	.16
			Max	32.05	±3.67	21.80	±3.59	.03

ROM, range of motion.

Table II shows only categories with significant results (*P* < .05).

### I shifting

For the wrist, the statistical analysis revealed a significant smaller ROM for the right wrist extension when driving with the right restricted elbow (free ROM: −8.75° to −44.10° vs. elbow restriction: −15.70° to −39.00° extension; *P* = .02), but no significant difference for the left wrist was found.

For the elbow, the statistical analysis revealed a significant smaller ROM for the right elbow pronation or supination (free ROM: 72.15°-113.80° vs. elbow restriction: 78.15°-91.10° (pronation (>90°) or supination (<90°)); *P* = .09) but for the left elbow, a significant higher supination (free ROM: 77.75°-92.90° vs. elbow restriction: 68.10°-92.00° (pronation (>90°)/supination (<90°)); *P* = .09) and flexion (free ROM: 44.60°-56.80° vs. elbow restriction: 46.75°-81.35°; *P* < .01) when driving with the right restricted elbow was found.

For the shoulder, the statistical analysis revealed a significant higher ROM for abduction or adduction (free ROM: 10.60°-29.70° vs. elbow restriction: 15.75°-46.55°; *P* < .02) with a shift to a higher mean abduction (mean abduction: free ROM: 18.46° vs. elbow restriction: 26.74°; *P* < .01), a significant higher ROM for internal rotation (free ROM: −3.90° to 17.65° vs. elbow restriction: −24.10° to 14.80° (external rotation (+)/internal rotation (−)); *P* < .01), with a shift to a higher mean internal rotation (mean internal rotation/external rotation: free ROM: 8.84° vs. elbow restriction: −2.90°; *P* < .01) and a significant higher ROM for flexion (free ROM: 22.50°-47.55° vs. elbow restriction: 11.40°-47.85°; *P* < .01) for the right shoulder when driving with the right restricted elbow. Moreover, for the left shoulder, a significant higher ROM for abduction or adduction (free ROM: 1.75°-8.10° vs. elbow restriction: 5.25°-16.20°; *P* = .03) with a shift to a higher mean abduction (mean abduction: free ROM: 4.46° vs. elbow restriction: 9.56°; *P* < .01) and a significant higher ROM for external rotation (free ROM: 16.15°-24.60° vs. elbow restriction: 8.25°-23.50° (external rotation (+)/internal rotation (−)); *P* < .01), with a shift to a lower mean external rotation (mean external rotation: free ROM: 21.19° vs. elbow restriction: 16.41°; *P* < .01) was found when driving with the right restricted right elbow.

### II left turns

For the wrist, the statistical analysis revealed a significant smaller ROM for the right wrist extension when driving with the right restricted elbow (free ROM: −3.80° to −44.70° vs. elbow restriction: −5.25° to −35.75° extension; *P* < .01), but no significant difference for the left wrist was found.

For the elbow, the statistical analysis revealed a significant smaller ROM for the right elbow pronation or supination when driving with the right restricted elbow (free ROM: 46.60°-125.25° vs. elbow restriction: 80.05°-95.15° (pronation (>90°)/supination (<90°)); *P* = .03), but no significant difference for the left elbow was found.

For the shoulder, the statistical analysis revealed a significant smaller ROM for abduction or adduction (free ROM: −3.90° to 17.95° vs. elbow restriction: 9.05°-22.85° (abduction (+)/adduction (−)); *P* < .01), with a shift to a higher mean abduction (mean abduction: free ROM: 5.36° vs. elbow restriction: 16.14°; *P* < .01) and a significant smaller ROM for flexion (free ROM: 28.20°-64.00° vs. elbow restriction: 33.55°-62.35°; *P* = .04) for the right shoulder when driving with the right restricted elbow. No significant difference for the left shoulder was found.

### III right turns

For the wrist, the statistical analysis revealed a significant smaller ROM and shift for the right wrist to a lower extension when driving with restricted elbow (free ROM: −8.25° to −48.60° vs. elbow restriction: −1.45° to −35.15° extension; *P* = .01), but no significant difference for the left wrist was found.

For the elbow, the statistical analysis revealed a significant smaller ROM for the right elbow pronation or supination when driving with the right restricted elbow (free ROM: 45.20°-110.45° vs. elbow restriction: 74.55°-91.20° (pronation (>90°)/supination (<90°)); *P* = .03), but no significant difference for the left elbow was found.

For the shoulder, the statistical analysis revealed a significant smaller ROM for abduction or adduction (free ROM: 4.20°-22.25° vs. elbow restriction: 12.90°-26.15°; *P* < .01) with a shift to a higher mean abduction (mean abduction: free ROM: 12.22° vs. elbow restriction: 18.81°; *P* < .01) and a significant smaller ROM for external rotation (free ROM: −0.05° to 32.05° vs. elbow restriction: −3.95° to 21.80° (external rotation (+)/internal rotation (−)); *P* = .03) for the right shoulder when driving with the right restricted elbow. No significant difference for the left shoulder was found.

## Discussion

This study analysis driving performance and biomechanical changes of movement patterns of the wrist, elbow, and shoulder, when driving with restricted flexion and extension of the right elbow.

The functional motion arc of the elbow used when driving was described in a previous study and lies between 5° to 105° flexion and 45° to 35° pronation or supination.^17^ Previous studies also suggest that movement restriction of the elbow impairs driving performance.^29^ However, no study exists that analyzes biomechanical changes of movement pattern when driving with the restricted elbow joint. Therefore, it is not known, which driving maneuver can be compensated by which adjacent joint. Moreover, there is a lack of evidence about which degree of freedom of the elbow ROM, pronation or supination or flexion or extension, is more important for driving performance and compensation capability. Thus, in this study, only flexion or extension of the elbow was restricted and analyzed. A further study is planned to evaluate the impact of pronation or supination on driving and compensation capability.

### Driving performance

To evaluate driving performance, speed, lane accuracy, and shifting time was measured when driving with free ROM versus when driving with the right elbow restricted in 90° flexion but with free pronation and supination. Our results showed, that participants drove their car in a significant lower maximum speed (37.45 ± 1.66 km/h vs. 32.53 ± 1.18 km/h; *P* = .02) while driving right turns with the right elbow restriction. In contrast, elbow restrictions had no significant effects on driving performance when driving left turns and when changing gears while driving straight on. Our results suggest that especially for right-handed people, compensation of right turns with the restricted right elbow ROM seems to be critical, and participants had to drive their car significantly slower to keep the lane accuracy constant. Our findings are in line with previous studies that suggest that movement restriction of the elbow impairs the driving performance.^12^^,^^29^ Especially in forensic medicine or for accident analysis, our detailed results could be of greater interest: driving straight on, changing gear, and left turns can be compensated without any loss of performance, but right turns are critical.

However, this study does not investigate possible adaptive processes and motor learning effects that occur if participants get familiar with compensation mechanisms. When evaluating compensation capability while driving in a new condition (restricted right elbow), participants will automatically start to learn in a trial-and-error process.^25^ To minimize learning effects, every course and maneuver were carried out as short as possible, and each maneuver and condition was driven in randomized order. However, especially for people with permanent functional impairment, mid- and long-time learning and adaptive processes could be of great interest. Further studies with a different experimental setup are needed to evaluate mid- and long-term motor learning effects when driving impaired. This study focuses on the short-term compensation mechanism of restricted elbow ROM, as it is due to cast immobilization, sprain, or fracture.

Defining driving performance is an important aspect of this study. In fact, defining driving performance exclusively is impossible, and sufficient surrogate parameters are still discussed controversially.^19^ For lower extremities, well-established surrogate parameters are breaking reaction time (approximately 750 ms) and brake force (approximately 100 NM).^3^^,^^4^^,^^7^^,^^14^^,^^19^^,^^32^^,^^20^ In contrast, for upper extremities, there is a wide inventory of possible surrogate parameters due to movement complexity, ranging from steering reaction time, lap time, number of collisions with cones, and lane accuracy.^10^^,^^12^^,^^21^^,^^28^ In line with previous studies, driving performance was defined using the parameters lane accuracy, speed, and time of changing gear. To analyze complex movements of the upper extremity in detail, driving maneuvers were chunked into short and fundamental maneuvers (left turn, right turn, and changing gear).

### Movement pattern

The upper extremity works as a functional unit consisting of the wrist, elbow, and shoulder as a highly variable and adaptive organ for manipulating.^26^^,^^36^ Especially movement restrictions of the elbow result in a significant impairment when executing daily tasks and seem to force the adjacent joints (wrist and shoulder) into a highly different movement pattern.^24^ Driving can be defined in a number of driving-related activities.^11^ Arms are mainly involved in steering wheel and shifting gear. Our driving performance results (Table II) suggest that driving straight on and changing gear and driving left turns can be compensated while driving with restricted right elbow flexion or extension, and continuing driving in a compensated way is possible. However, our movement pattern results show how compensation is made on biomechanical level.

Combination of shifting gear and holding the steering wheel is a bimanual task. Our movement pattern results suggest that shifting gear while driving straight on with restricted right elbow is compensated with a different movement pattern of the left elbow and both shoulders. Left elbow is mainly used in a higher supination and flexion, left shoulder in a higher abduction. This posture allows the left arm to hold the steering wheel close to the body, particularly firmly and controlled, while operating the gear in a new way with the right arm. This is in line with previous studies that show an increased muscle activation of biceps when the upper arm is abducted and the forearm is supinated.^22^^,^^23^ While holding the steering wheel firmly with the left arm, loss of extension of the right elbow is compensated by the right shoulder in an increased internal rotation, abduction, and flexion to operate the gear lever.

Combination of shifting gear and steering wheel is a bimanual task. In contrast, when steering the wheel solely, hands often operate in a redundant way, positioned in a 10 and 2 o’ clock position, where one hand is often dominant and the other hand only assists.^13^ Previous studies suggest that hand position patterns vary with speed and complexity of the driving environment.^35^ While driving complex roads, a two-handed style is preferred, and while driving simple roads, one hand is dominant and the other hand assists.^35^ Our data of driving left turns suggest that left turns can be well compensated with a posture chance of the right shoulder with a shift to higher abduction while the left arm is used in the same way. Our data further suggest that compensation of right turns is critical with a posture chance of the right shoulder with a shift to higher abduction while left arm is used in the same way.

While changing gear and left turns can be compensated, drivers should be aware when driving right turns and especially when driving turns in combination with changing gears, which cannot be done in a bimanual and arm-redundant driving style.^35^ Attending physicians should pay special attention to patients ROM of both shoulders and contralateral elbow, when evaluating the driving capability of patients who had a short-term movement restriction of the right elbow due to a cast immobilization, sprain, or fracture.

### Limitations

This study has several limitations. We took into account that, when evaluating driving fitness, we need to address different car types. Approximately 3% of cars sold in the United States. were built with manual transmissions.^5^ In contrast, 80% of cars sold in Europe were built with manual transmissions.^6^ Driving can be defined as a finite number of driving-related activities.^11^ Arms are mainly involved in steering wheel and shifting gear. To evaluate biomechanical parameters for manual and automatic transmission very precisely, participants drove turns in automatic mode, and shifting gear was evaluated separately.

In this study, only right-handed and healthy subjects were included, and movement restrictions were simulated using orthosis. In contrast, in several previous studies, *return-to-drive* time was evaluated based on different orthopedic diseases, injuries, or postoperative conditions.^9^^,^^31^^,^^33^^,^^34^ However, our experimental setup with healthy participants and simulated limitations was chosen deliberately. It is not expedient to evaluate *return-to-drive* time based on orthopedic diseases, injuries, or fractures. Even if similar fracture morphologies exist in the usual places, symptoms after treatment can range from complete functional preservation to complete loss of function of a joint.^30^^,^^33^ Due to this high variance in the field of orthopedics and trauma, *return-to-drive* time is still discussed controversially.^33^ Therefore, it seems more effective, to evaluate *return-to-drive* time based on functional parameters, like ROM of one joint. Moreover, parameters like ROM can easily and objectively be checked by attending physicians to evaluate driving fitness. Yet, there do still exist little evidence-based data on functional biomechanics necessary to drive a car safely.^15^, ^16^, ^17^, ^18^, ^19^ This study adds new evidence-based functional biomechanical parameters that can help attending physicians when evaluating driving fitness.

## Conclusion

This study first describes changes in movement patterns of the upper extremity while driving with a restricted right elbow. Our data suggest that restricted right elbow flexion or extension can be compensated by the left arm and leads to a different posture of the right shoulder when driving left turns. A different movement pattern of the left elbow and both shoulders is used, when changing gears while driving straight. Drivers should be aware when driving turns while shifting gears, and special attention should be paid to the shoulders and left elbow when evaluating the driving capability of patients with movement restriction of the right elbow by physicians.

## Disclaimers

Funding: This study was partially financed by Allgemeiner Deutscher Automobil-Club Stiftung.

Conflicts of interest: The authors, their immediate families, and any research foundation with which they are affiliated have not received any financial payments or other benefits from any commercial entity related to the subject of this article.
